# RETRACTION: MicroRNA‐520a Suppresses Pathogenesis and Progression of Non‐Small‐Cell Lung Cancer through Targeting the RRM2/Wnt Axis

**DOI:** 10.1155/ancp/9864065

**Published:** 2026-01-08

**Authors:** Analytical Cellular Pathology

RETRACTION: Y. Xie, C. Xue, S. Guo, and L. Yang, “MicroRNA‐520a Suppresses Pathogenesis and Progression of Non‐Small‐Cell Lung Cancer through Targeting the RRM2/Wnt Axis,” *Analytical Cellular Pathology* 2021, no. 1 (2021): https://doi.org/10.1155/2021/9652420.

The above article, published online on 31 March 2021 in Wiley Online Library (wileyonlinelibrary.com), has been retracted by agreement between the journal Editor‐in‐Chief, Dimitrios Karamichos and John Wiley & Sons Ltd. The retraction has been agreed after an investigation into concerns raised by *Hoya camphorifolia*, *Indigofera tanganyikensis* and *Aphilanthops foxi* via PubPeer [1].

Specifically:

- Unexpected similarities were found between Figure 5D (panel H460 – miR‐520a control) and Figure 5G, panel (HL‐60 – ZNF667‐AS1 si + miR‐206 inh) in [2];
- Incorrect measuring of necrotic cells as apoptotic cells in Figure 2E and Figure 4E;
- The primers used in this study were found to recognize the Human miR‐506 precursor molecule, instead of the reported miR‐520a. The selected target, RRM2, also appears to be an unsuitable choice for targeting miR‐520a‐3p.

Multiple attempts by the journal to contact the authors have not resulted in a reply. As such, the Chief Editor considers the conclusions of this article to be invalid.
